# Investigating the Use of Electrooculography Sensors to Detect Stress During Working Activities

**DOI:** 10.3390/s25103015

**Published:** 2025-05-10

**Authors:** Alessandra Papetti, Marianna Ciccarelli, Andrea Manni, Andrea Caroppo, Gabriele Rescio

**Affiliations:** 1 Department of Industrial Engineering and Mathematical Sciences, Università Politecnica delle Marche, 60131 Ancona, Italy; m.ciccarelli@staff.univpm.it; 2 National Research Council of Italy, Institute for Microelectronics and Microsystems, Via Monteroni, c/o Campus Ecotekne, Palazzina A3, 73100 Lecce, Italy; andrea.manni@cnr.it (A.M.); andrea.caroppo@cnr.it (A.C.); gabriele.rescio@cnr.it (G.R.)

**Keywords:** electrooculography, human factors, smart eyewear, stress detection, supervised machine learning, random forest

## Abstract

**Highlights:**

**What are the main findings?**

- EOG signals from smart eyewear effectively detect stress in controlled tasks
- Random forest outperforms with 85.8% (2-class) and 82.4% (3-class) accuracy

**What is the implication of the main finding?**

- Smart glasses may enable practical stress detection in real-world work settings
- Future work should explore real-world settings and multimodal signal inputs

**Abstract:**

To tackle work-related stress in the evolving landscape of Industry 5.0, organizations need to prioritize employee well-being through a comprehensive strategy. While electrocardiograms (ECGs) and electrodermal activity (EDA) are widely adopted physiological measures for monitoring work-related stress, electrooculography (EOG) remains underexplored in this context. Although less extensively studied, EOG shows significant promise for comparable applications. Furthermore, the realm of human factors and ergonomics lacks sufficient research on the integration of wearable sensors, particularly in the evaluation of human work. This article aims to bridge these gaps by examining the potential of EOG signals, captured through smart eyewear, as indicators of stress. The study involved twelve subjects in a controlled environment, engaging in four stress-inducing tasks interspersed with two-minute relaxation intervals. Emotional responses were categorized both into two classes (relaxed and stressed) and three classes (relaxed, slightly stressed, and stressed). Employing supervised machine learning (ML) algorithms—Random Forest (RF), Logistic Regression (LR), Support Vector Machine (SVM), Decision Tree (DT), and K-Nearest Neighbors (KNN)—the analysis revealed accuracy rates exceeding 80%, with RF leading at 85.8% and 82.4% for two classes and three classes, respectively. The proposed wearable system shows promise in monitoring workers’ well-being, especially during visual activities.

## 1. Introduction

Work-related stress has become a pressing concern in modern workplaces, contributing to decreased productivity, increased absenteeism, and serious health consequences such as burnout, anxiety, and cognitive overload. As industries become increasingly digitized and interconnected, the need for effective stress-monitoring solutions becomes more urgent. At the same time, traditional physical labor is progressively being replaced by cognitively demanding tasks—such as decision making, supervision, and system control—which place a heavier load on workers’ mental resources. This shift underscores the importance of identifying and managing stress not only from a physical standpoint but also from cognitive and emotional perspectives.

In this context, Industry 5.0 emerges as a forward-looking paradigm that places the human worker back at the center of the industrial process. Unlike its predecessor (Industry 4.0), which focused on automation and data exchange in manufacturing technologies, Industry 5.0 emphasizes human centricity, sustainability, and resilience. This shift requires organizations to not only integrate advanced technologies but also actively support the mental and emotional well-being of their workforce.

To meet these new demands, organizations must adopt a comprehensive strategy that supports the integration of technology while also addressing psychological challenges associated with evolving work environments, such as cognitive overload, reduced attention, emotional fatigue, and burnout. This includes offering targeted interventions such as adaptive workload redistribution, job rotation, mental health support, and biofeedback-based relaxation techniques, supported by real-time data collection.

Within this framework, sensor-based and vision-based techniques can play a significant role in monitoring and managing stress levels in the workplace. These techniques utilize physiological measurements to gather data on various aspects of human health and well-being.

Electrocardiogram (ECG) and electrodermal activity (EDA) features are physiological measurements that are widely used for monitoring work-related stress. On the other hand, eye movement patterns can also provide insights into cognitive processes, attention, and mental workload, which are all factors relevant to work-related stress. These patterns are primarily examined through vision-based sensors, known for their cost effectiveness and minimal need for intervention in configuration and setup. Stress assessment via non-wearable sensors is a focal point in the scientific literature, spanning diverse application areas. For instance, Marcos-Ramiro et al. [1] proposed a method for automatically detecting blinks in video sequences of conversations with the aim of discovering stress. Specifically, the authors performed per-pixel classification of the extracted eye images to detect blinks through their dynamics. The system’s performance was evaluated using a job interview database with annotations of psychological variables, demonstrating a statistically significant correlation between perceived stress resistance and automatically detected blink patterns.

Mohd et al. [2] proposed an integrated non-invasive measurement through purely imaging means. The objective of the work was to use vision-based measurement as an alternative to physiological measurement. The proposed research consisted of three vision features: eye blinking from a visual sensor, skin temperature from three ROI’s, and blood vessel volume at the supraorbital from a thermal IR camera. The results reported in this work were very promising, although a limitation arose from the use of features extracted from IR images, for which the acquisition device is not low cost, making this solution less widely deployable in a working context.

On the other hand, Korda et al. [3] investigated eye behaviors through blink activity during stress conditions. The authors used eye aperture time series and corresponding eye-related features, such as eye blinks and relative duration, extracted from facial videos, to discriminate between stressed and neutral tasks. They employed Convolutional Neural Networks (CNNs) and Long Short-Term Memory (LSTM) network models, achieving an average accuracy of about 81%, which was very promising given the unimodal analysis and the non-invasive modality used for stress evaluation.

A very recent work, specifically focused on assessing stress in the workplace, considers heart rate and galvanic skin response retrieved from a wearable device but used in conjunction with features extracted from the eye and processed through a consumer passive vision sensor [4]. Through image processing methodologies, eye tracking was performed, and the ratio (which identified the eye’s aperture width) was calculated by dividing the lengths of two axes of the eyes (vertical and horizontal). Next, eye blink was calculated by comparing the ratio value with a specific threshold value, estimated in the calibration phase of the entire system. Finally, the number of blinks was estimated within a given time window for the classification of stress levels, utilizing machine learning techniques, both supervised and unsupervised.

Electrooculography (EOG), which measures the electrical activity generated by the muscles responsible for eye movement, has been less studied in the context of work-related stress monitoring. However, it holds potential for similar purposes.

EOG is a valuable technology primarily utilized for eye tracking and diagnosing eye-related disorders. However, its potential goes beyond medical applications. Research has shown that EOG signals can significantly enhance communication abilities and improve the quality of life for individuals with disabilities [5,6].

One remarkable advancement in this field is the development of EOG-based interfaces designed for human–computer interaction. These interfaces enable individuals to control various devices by utilizing their eye movements as input commands. For instance, EOG signals have been used to develop a virtual keyboard [7], to control computer commands for navigation purposes [8,9] and to control reach-to-grasp movements of a robot arm [10], enhancing communication abilities for disabled individuals. By capturing and analyzing the electrical signals generated by the eye muscles, EOG-based systems accurately track eye movements, allowing individuals to interact with technology in an intuitive and accessible manner.

EOG offers several advantages, including linearity, non-invasiveness, affordability, and unobtrusive monitoring that does not interfere with vision. Although EOG may have lower spatial and temporal resolution compared to other eye-tracking methods, like video oculography or infrared oculography, it compensates with its cost effectiveness and the capability to capture eye movements even when the eyes are closed [11,12]. This makes EOG a valuable tool for monitoring eye-related behaviors and mental health, especially in mobile settings and long-term recordings [13]. In dimensional emotion recognition, EOG is frequently incorporated alongside other signals to enhance classifier performance, as it offers complementary features [14].

It is important to note that EOG signals can be affected by noise and share the same frequency band as the noise. Researchers have proposed various denoising techniques to address this challenge and improve the quality of EOG signals for analysis [15].

To implement EOG effectively, the placement of electrodes is also crucial. Traditionally, electrodes are positioned near the eyes to capture the electrical signals generated by eye movements and involve long wires for the connection to the data logging unit that restrict the user’s movements and introduce power-line interferences and motion artifacts during measurements [16]. Researchers have also explored alternative electrode placement, such as the forehead, to achieve a more practical and reliable acquisition of EOG signals [17]. However, in the context of using EOG in a working environment, it is important to address these issues and consider less invasive solutions. This is particularly relevant to ensure comfort and ease of use for individuals during extended periods of work. One such solution is the integration of EOG technology into wireless systems such as glasses or other wearable devices. Few works have foreseen the use of these solutions. For example, Lagodzinski et al. [18] used a dataset obtained with JINS-MEME glasses and employed a machine learning algorithm like a codebook approach to address the uncertainty of which features were effective for cognitive activity recognition. Rather than relying on feature engineering, this method utilized a distribution of characteristic subsequences (codewords) to describe recorded EOG data sequences.

Activity recognition is one of the main uses of EOG in a work context, along with the detection of drowsiness and fatigue. The main objective is to minimize the risk of injuries caused by fatigue and tiredness, particularly in the transport sector [19,20]. For example, studies have explored the relationship between fatigue and various eye metrics, such as pupil-based, blink-based, and saccade-based measurements. These investigations have shown that combining multiple eye metrics, along with camera-based eye-tracking systems, can provide a promising solution for fatigue detection in transportation settings. However, it is important to recognize the distinction between drowsiness and other types of fatigue, such as mental fatigue, as their physiological indicators may differ [21].

Furthermore, analyzing eye movements has become an innovative sensing modality for activity recognition. EOG-based algorithms have been successfully used to recognize various office activities, such as reading, writing, and resting [22,23].

In terms of fatigue detection in office environments, studies have examined features such as blink duration, blink amplitude, and time between blinks. The analysis of these features during tasks inducing fatigue, such as the N-back task, has shown a decrease in time between blinks and blink amplitude and an increase in blink duration, indicating the presence of fatigue [24].

Research suggests that the majority of information acquisition occurs through visual channels, implying that the analysis of eye blinking patterns can provide insights into the cognitive demands of a task. It is theorized that increased alertness and focused attention result in longer intervals between blinks, which serve as an indicator of heightened mental workload. The blink interval increases significantly with the difficulty of a tracking task but not with an arithmetic task. It appears that arithmetic tasks are not suitable for assessing resource limitations based on blink interval alone [25].

Eye dynamics, including blink duration, blink rate, and saccade characteristics, have been found to be influenced by mental activities. The number of saccades has been shown to decrease with increasing heart rate during cognitively stressful situations. Eye parameters have proven to be effective in classifying different stress stages, and their combination with heart rate variables has further improved classification accuracy [26].

However, most existing research on stress detection fails to take into account the measurement of EOG [27]. This article seeks to rectify this limitation by analyzing the EOG signal as a potential indicator of stress. To accomplish this, JINS MEME glasses equipped with sensors capable of capturing EOG signals are used. These glasses allow for non-intrusive and convenient data collection, ensuring a more natural and realistic assessment of stress in real-world scenarios. Furthermore, well-established protocols for stress induction typically used in laboratory experiments are employed. Understanding the role of EOG in stress detection can pave the way for more accurate and timely interventions, enhancing individual well-being and performance in high-stress environments. There is a scarcity of research on the utilization of wearable sensors in the field of human factors and ergonomics (HF/E), particularly when it comes to assessing human work [28]. This work aims to help address this gap.

## 2. Materials and Methods

In this experimental study, 12 volunteers (6 males and 6 females) participated, ranging in age from 22 to 39 (mean age = 28.08 years).

Before the test began, the room was prepared with the necessary equipment, and the devices were set up to avoid technical problems during the experimentation. User data were collected, and participants were briefed on the test without task instructions. Consent forms were signed, phones were turned off, and the JINS MEME glasses were worn.

**Figure 1:**
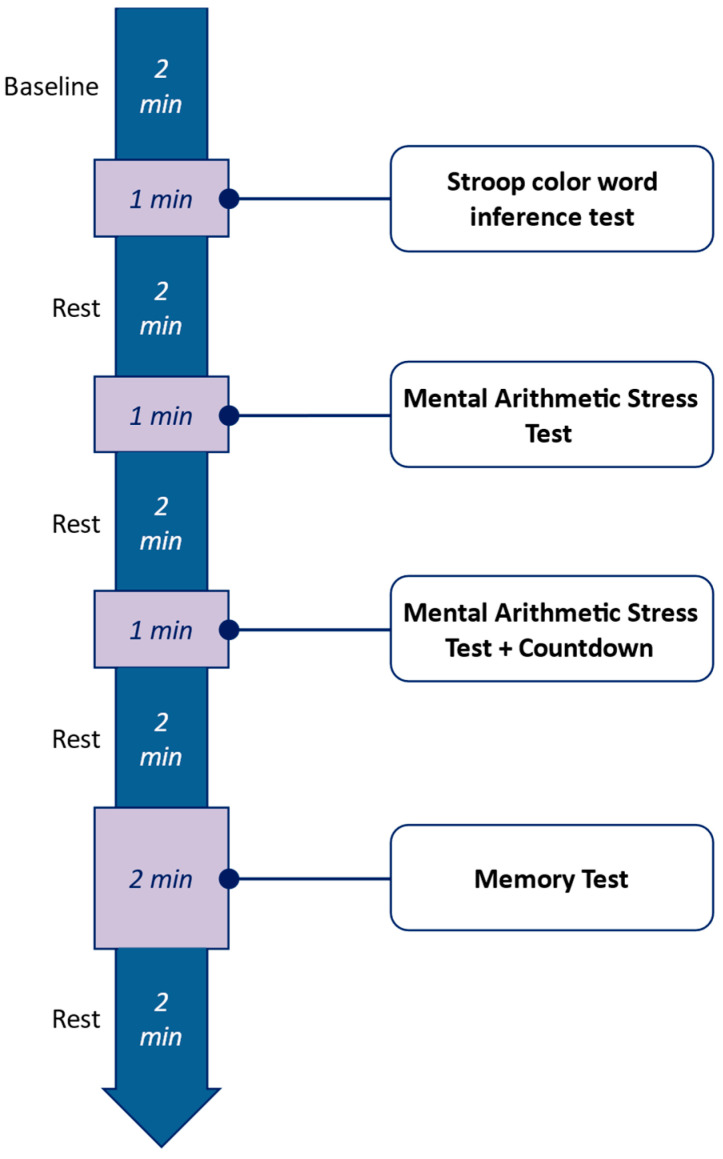
Experiment procedure.

The procedure of the experiment is illustrated in Figure 1. Participants were instructed to perform four stress-inducing tasks. Task instructions were displayed on a screen to minimize moderator interaction. Each phase is briefly described below.

To establish a baseline, participants underwent a 2 min resting period during which no stimuli were presented. During this time, they were instructed to relax while the experimenters temporarily left the room.

Task 1 involved the classic Stroop Color-Word Test (SCWT) [29], a well-established method for inducing mild cognitive stress. Participants were presented with color words printed in incongruent ink colors and instructed to name the ink color aloud. This task leverages the cognitive conflict between automatic word reading and controlled color naming.

Task 2 consisted of a mental arithmetic task requiring participants to count backward from 4000 in decrements of 7. To increase the pressure, incorrect responses triggered an audible alert, prompting participants to correct their mistakes. While this task has been shown to minimally affect blink intervals [21], it was included to evaluate its potential influence on cognitive load.

Task 3 was a modified version of Task 2, introducing a time constraint to heighten stress levels. A visual countdown timer was displayed on the screen, adding time pressure to the mental arithmetic calculations.

Task 4 was a memory recall task in which participants were presented with a 6 × 6 grid of numbered cards. After a brief viewing period (30 s), the cards were concealed, and participants were asked to recreate the sequence by clicking on the cards in the correct order. Incorrect selections resulted in a reset, increasing the pressure to perform accurately and efficiently.

**Figure 2:**
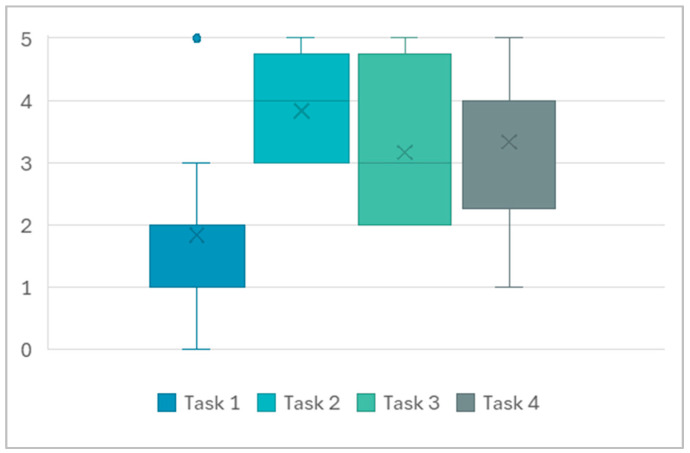
Perceived cognitive load per task.

Upon completing all tasks, participants rated their perceived cognitive load for each task using a 5-point Likert scale, with 0 representing “no stress at all.” As illustrated in Figure 2, the SCWT generally induced lower perceived stress compared to the other tasks. Mental arithmetic tasks, particularly under time pressure, emerged as a more potent stressor for most participants. However, the memory recall task showed greater individual variability in perceived stress levels.

### 2.1. Data Collection

**Figure 3:**
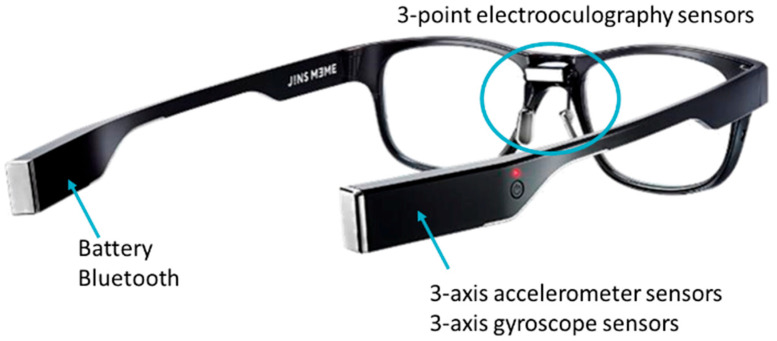
JINS MEME ES_R glasses.

For data collection purposes, participants were instructed to wear JINS MEME ES_R glasses (Figure 3), which belong to the category of intelligent eyewear developed by JINS, a Japanese eyewear company. These glasses seamlessly integrate traditional eyeglasses with advanced sensor technology to offer a wide range of features and functionalities. The glasses are equipped with built-in sensors, including EOG sensors for precise eye movement detection, a 3-axis accelerometer for motion sensing, and an optical sensor for accurately measuring brightness levels. Additionally, the glasses come with a lightweight and durable frame design, ensuring comfort for extended wear.

The EOG signal was sampled at a rate of 50 Hz, which is sufficient for detecting key eye movement features such as blinks and slow-phase dynamics relevant for stress and fatigue analysis. Data were transmitted wirelessly via Bluetooth 4.0 Low Energy (BLE) from the glasses to a connected PC, where they were stored locally for subsequent analysis. The real-time acquisition allowed for immediate verification of signal quality during the experimental session. To ensure accurate alignment between physiological signals and experimental tasks, timestamping was used to mark the onset and progression of each task segment.

**Figure 4:**
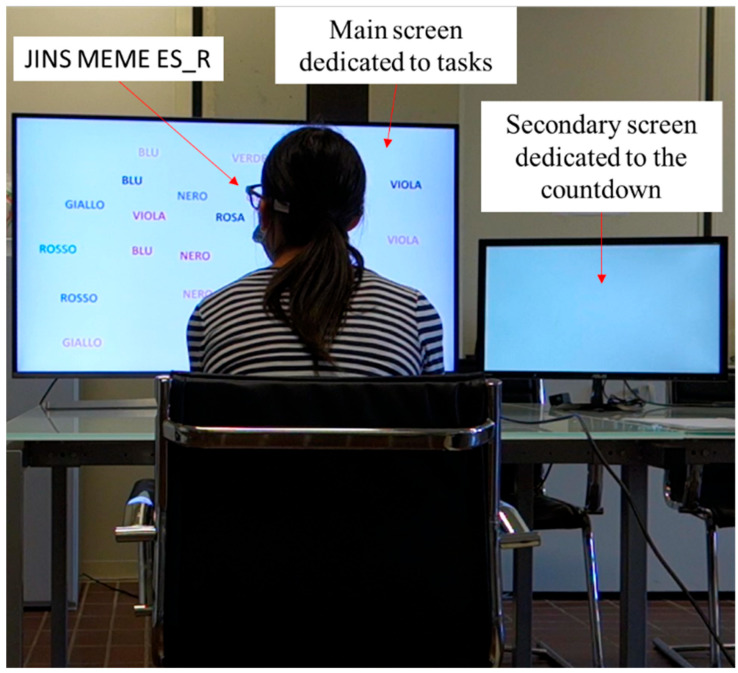
User performing the Stroop Color-Word Test.

Figure 4, illustrating the laboratory arrangement, displays a participant wearing JINS MEME ES_R glasses, engaged in the execution of Task 1, specifically the Stroop Color-Word Test.

### 2.2. Pre-Processing

The goal of this step was to minimize baseline noise, environmental artifacts, and signal artifacts caused by potential movements of the glasses. This was accomplished by applying a 6th-order Butterworth bandpass filter with a frequency range of 0.25 Hz to 7.5 Hz to the acquired signal, which was initially sampled at 50 Hz. Additionally, acceleration data from the glasses were analyzed to address artifacts resulting from sudden head movements or disruptions in electrode contact with the skin on the nose. In particular, acceleration data were preprocessed by applying an 8th-order low-pass FIR filter with a cutoff frequency of 10 Hz, which was designed to attenuate noise components from electronic circuitry, environmental interferences, and physiological tremors. Then, the magnitude of the 3D acceleration vector was computed and a threshold was defined as the mean plus three times the standard deviation of the acceleration magnitude. As a result, EOG peak signals associated with excessively high acceleration values were eliminated.

**Figure 5:**
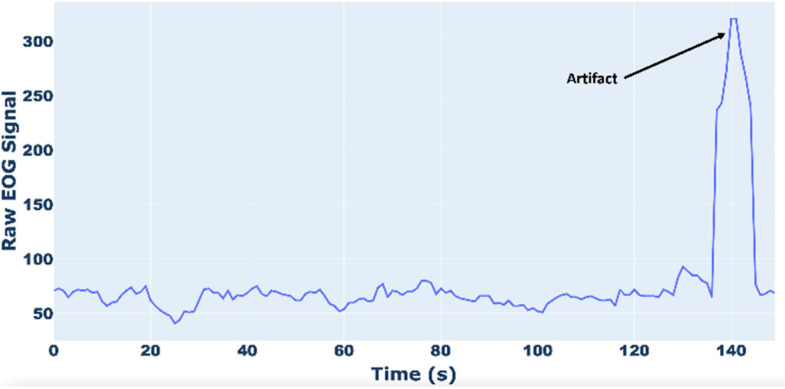
Example of raw EOG signal.

**Figure 6:**
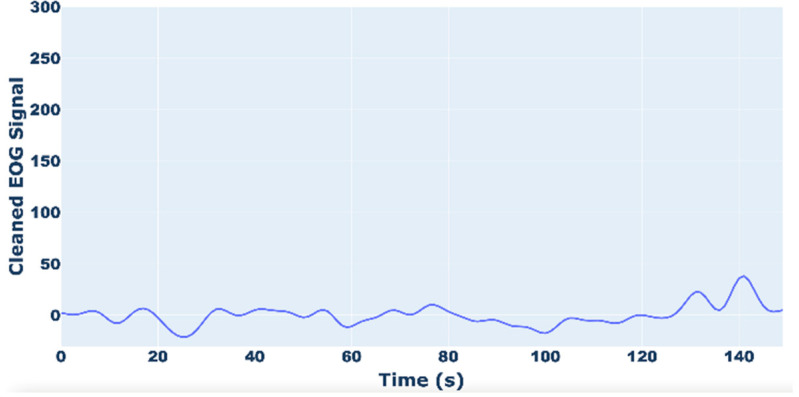
Example of cleaned EOG signal.

In Figure 5 and Figure 6, examples of raw and filtered EOG signals are reported. Figure 6 shows how the peak of the EOG signal, due to artifacts, is eliminated.

### 2.3. Feature Extraction and Selection

The objective of the feature extraction phase was to identify and retrieve pertinent information from the EOG signal with the aim of discerning potential stress events experienced by the end user. The features were extracted using a sliding window of three seconds with a step of 1 s, resulting in 882 samples per user.

In particular, the calculated features are reported in Table 1.

Eye blinks were detected from the vertical EOG signal using the NeuroKit2 (vers. 0.1.7) Python library. This method relies on a template-matching approach, where candidate blink events are identified based on their similarity to an optimized template function. For each detected event, a root mean square error (RMSE) is computed between the event and the template, and events with low RMSE values are classified as blinks according to an adaptive threshold.

To reduce the complexity of signal processing and increase the performance of the system, the Lasso algorithm [30] was applied to select the most effective subset of EOG features. This algorithm was implemented due to its demonstrated efficacy in supervised algorithmic contexts.

Following the application of the Lasso algorithm to the aforementioned feature set, the mean and standard deviation of the total EOG signal were removed.

### 2.4. Classification

Following the aforementioned methodology, the data were classified into two categories (stress/no stress) and three categories (no stress, stress level 1, and stress level 2) based on the participants’ self-reported stress levels. Subsequently, five machine learning (ML) supervised algorithms were trained on these data for comparison purposes. The considered classifiers were: Random Forest (RF), Logistic Regression (LR), Support Vector Machine (SVM), Decision Tree (DT), and K-Nearest Neighbors (KNN).

RF [31] employs predictors that are randomly generated from the dataset utilized. In particular, the classifier is automatically provided by an independent vector derived from the input vector and each generated tree is assigned the highest number of classes. The addition of further trees introduces further randomness into the model. The optimal feature is then identified in a randomly selected subset of features. With regard to the model parameters, the number of estimators in the forest was set to 8, while the maximum tree depth was set to 12.

The LR [32] algorithm is a significant contributor to machine learning, offering the ability to assign probabilities and rankings to new data sets, whether continuous or discrete. To achieve accurate classification, a well-known logistic function, known as a sigmoid function, is employed. This function represents the probability of a given outcome, such as the likelihood of cells being cancerous or not, or the probability of a user being stressed or not.

SVM [33] is a classification method that has been demonstrated to exhibit superior accuracy compared to other classifiers in a multitude of application domains. A hyperplane in N-dimensional space (where N is the number of features) is identified with the objective of maximizing the distance between each dataset and the baseline. The most representative observations for each reference class are designated as support vectors. An important parameter for the SVM is the kernel, which can be linear, polynomial, or radial. In this work, a linear kernel was employed.

DT [34] is a popular supervised ML algorithm in which data are partitioned on a given parameter. A tree is applied as a predictive model to arrive at the target value of the feature (represented by the leaves) crossing the observations (represented by the branches of the tree). In particular, the leaves are the class labels and the branches depict the feature junctions arising in the class labels. In our tests, the maximum depth of the tree was set at 15.

The KNN [35] classification method is straightforward to implement and exhibits high classification performance. The algorithmic approach involves assigning a sample to a class if most k neighboring samples belong to the same class. The optimal k value is crucial as it determines the extent to which the neighborhood may contain samples of other classes, or conversely, be affected by noise.

To identify the optimal parameters for each ML model, a grid search technique was employed [36]. The optimal parameters for two classes are presented in Table 2, while the optimal parameters for three classes are reported in Table 3.

## 3. Results and Discussion

To validate the proposed approach, a series of tests was implemented. The EOG signal acquisition and processing system was developed using the Python language and some packages such as Neurokit2, scikit-learn, etc. The tests were executed on an embedded PC with an Intel Core i5 processor and 8 GB RAM. The performance of the supervised classifiers was measured using four different metrics, accuracy (Acc), precision (Pr), recall (Re), F1 score, and specificity (Spe), which are specified by the following equations:

(1) $$Acc=\frac{Number\;of\;Correct\;Predictions}{Total\;Number\;of\;Predictions}=\frac{\sum_{i=1}^{C}{TP}_{i}}{\sum_{i=1}^{C}\left({TP}_{i}+{FP}_{i}{+FN}_{i}\right)}$$

(2) $${Pr}_{i}=\frac{{TP}_{i}}{{TP}_{i}+{FP}_{i}}$$

(3) $${Re}_{i}=\frac{{TP}_{i}}{{TP}_{i}+{FN}_{i}}$$

(4) $${F1-score}_{i}=\frac{2\;*\;{TP}_{i}}{2\;*\;{TN}_{i}+{FP}_{i}+{FN}_{i}}$$

(5) $${Spe}_{i}=\frac{{TN}_{i}}{{TN}_{i}+{FP}_{i}}$$

where *TP* (True Positive) indicates that the algorithm correctly detected a stress phase; *FP* (False Positive) indicates that the algorithm detected a stress phase that did not occur; *TN* (True Negative) indicates that the algorithm correctly identified the absence of a stress phase; and FN (False negative) implies that the algorithm did not detect a stress phase that did occur. Accuracy denotes the ratio of all correctly classified samples to all samples; precision shows the accuracy in predicting positive occurrences; recall shows the model’s performance in predicting positive occurrences using all positive cases; and the F1 score affects true positive cases more than precision.

The performance of each ML classifier was measured on randomly perturbed datasets by applying 10-way cross-validation [37]. So, each classifier was trained using 90% of the data, with the remaining 10% used for testing. Also, to prevent over-fitting, 10% of the training data was employed to create a validation set. Furthermore, the procedure was repeated 10 times, training the classifier with a different training set and testing it with a separate test set in order to avoid the same samples simultaneously appearing in the training and test sets.

**Figure 7:**
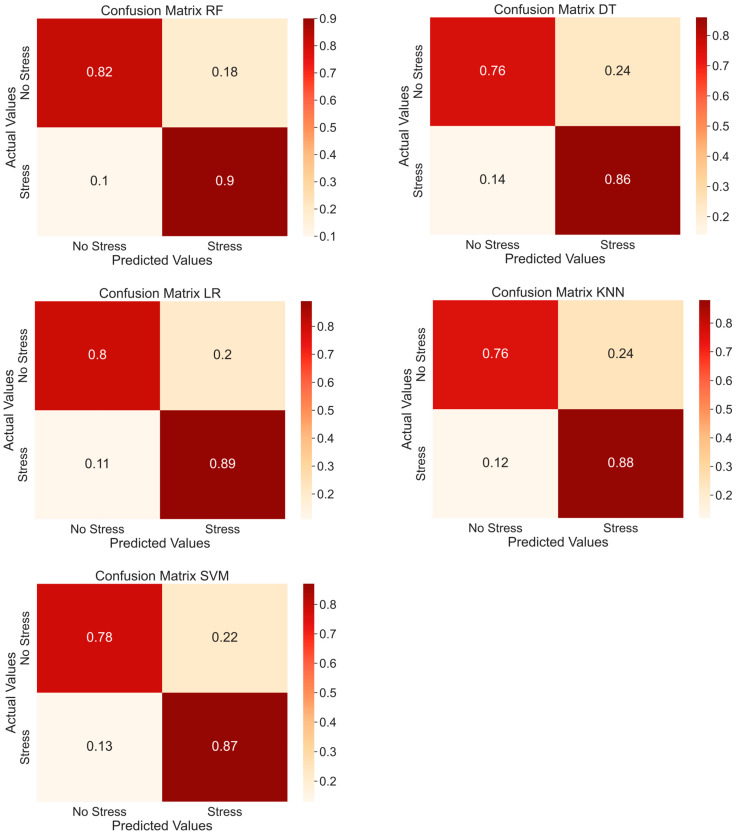
Confusion matrices using RF, LR, SVM, DT, and KNN as classifiers with two classes.

Table 4 presents the performance of each ML model considering two classes (stress/no stress), whereas in Figure 7 the confusion matrices of the average accuracies varying the considered classifiers are reported. The RF model showed the best performance in terms of average accuracy compared to the other classifiers. On the other hand, the DT model showed the worst performance. Specifically, RF had an improvement of about 4% in average accuracy compared to DT, with a value of 85.8% vs. 81%.

**Figure 8:**
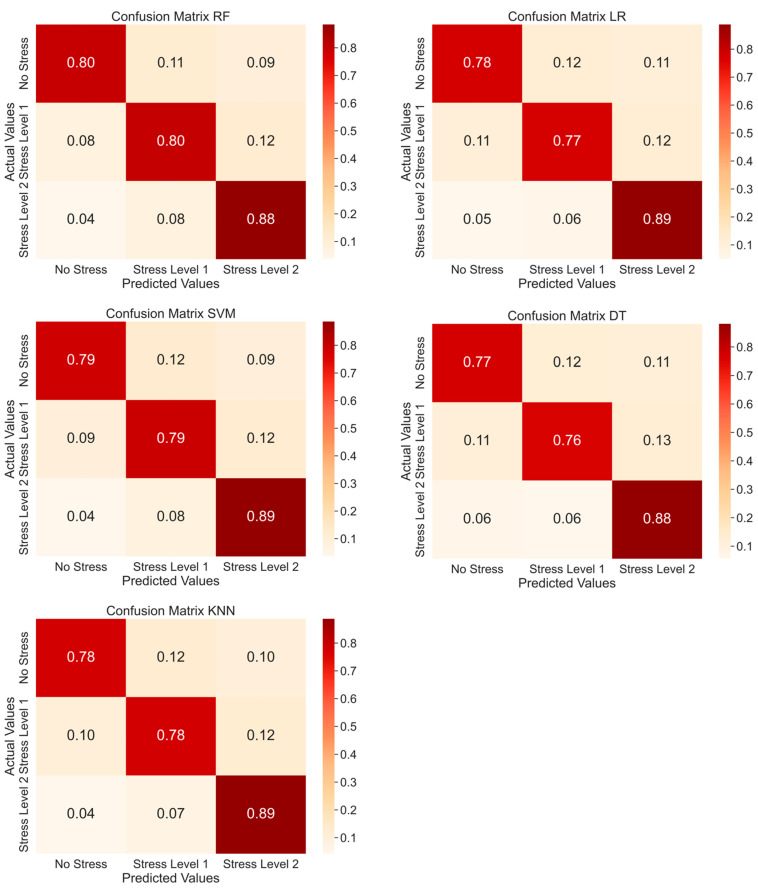
Confusion matrices using RF, LR, SVM, DT, and KNN as classifiers with three classes.

The performance of each ML model considering three classes (no stress, stress level 1, and stress level 2) is reported in Table 5, and the corresponding confusion matrices are presented in Figure 8. Again, RF exhibited the best performance compared to the other classifiers and DT showed the worst performance. In this case, the gap between the best and worst average accuracy was smaller (about 2%), ranging from 82.4% for RF to 80.2% for DT.

Furthermore, Table 6 and Table 7 show, for the best-performing classifier (RF), the average values of the considered metrics when varying the classes both with two classes and with three classes.

Furthermore, the performance of the best-performing classifier on the specific tasks was evaluated, and the average accuracy results are presented in Table 8. As can be seen, the classifier was able to classify Tasks 2, 3, and 4 with reasonable accuracy, while it was less accurate for Task 1, as Tasks 2, 3, and 4 differed more from the rest phase compared to Task 1.

Finally, to verify the models’ generalizability, the performance of the different considered classifiers was evaluated using leave-one-subject-out cross-validation. In this protocol, one subject was selected for testing and the remaining subjects served as the training set. In detail, the trials were replicated twelve times (the number of subjects in the dataset) and the average results over twelve times were reported in Table 9 and Table 10 and, as can be seen, they were consistent with those previously reported. Furthermore, the values of the considered metrics for the two and three classes, respectively, for the best performing classifier (RF) are displayed in Table 11 and Table 12.

## 4. Conclusions

This study explored the application of smart eyewear in stress detection, extracting crucial data from the EOG signal. Participants engaged in common stress-inducing tasks within a controlled environment, encompassing main working activities like reading, elaborating, calculating, and memory tasks. The experimental findings indicated that EOG features effectively discern stress conditions, with all five ML classifiers achieving accuracy rates exceeding 80%. The RF model outperformed others, boasting an accuracies of 85.8% and 82.4% for two classes and three classes, respectively.

Despite these promising results, the study presents some limitations. The relatively small and demographically homogeneous sample may limit the generalizability of the findings. Moreover, the experiment was conducted in a controlled and static setting, which helped to ensure signal stability but did not reflect the variability of real-world environments. The current study also focused on screen-based tasks, introducing a potential bias related to eye movement parameters. In future research, a different test protocol will be adopted to simulate non-screen-based working activities, with specific emphasis on visual inspection—an ideal task due to its reliance on sight.

No issues related to excessive sweating were observed during the tests; however, in dynamic or real-world contexts, sweat could impact signal quality and should be taken into account in future studies. Additional sources of noise and user-specific variability are also important aspects to address in more ecologically valid settings.

Exploring multimodal physiological sensing (e.g., combining EOG with HRV or EDA) and integrating real-time feedback systems could further enhance system robustness and user engagement.

A final practical consideration concerns the use of smart eyewear by individuals who already wear prescription glasses. While this does not compromise the validity of the current findings, it may limit usability and should be addressed in future research by exploring alternative wearable solutions capable of offering similar functionality.

## Figures and Tables

**Table 1:** Features extracted from the EOG signal.

Features
Frequency of eye blink
Mean of the peak-to-peak amplitude in the EOG vertical component
Variance of the EOG vertical component
Covariance of the vertical component
Standard deviation of EOG peaks
Root mean square error of the signal
Maximum of the EOG vertical component
Mean of the total EOG signal
Standard deviation of the considered signal

**Table 2:** Optimal parameters used for classification models with two classes.

Model	Parameters
RF	max_depth = 12
	n_estimators = 8
	criterion = gini
LR	solver = newton-cg
	max_iter = 75, C = 0.05
SVM	decision_function_shape = ovo
	max_iter = 80
	kernel = linear
	C = 0.1
DT	max_depth = 15
	criterion = gini
KNN	n_neighbors = 7
	metric = minkowski
	algorithms = auto
	weights = distance

**Table 3:** Optimal parameters used for classification models with three classes.

Model	Parameters
RF	max_depth = 14
	n_estimators = 6
	criterion = gini
LR	solver = newton-cg
	max_iter = 65, C = 0.03
SVM	decision_function_shape = ovo
	max_iter = 70
	kernel = linear
	C = 0.5
DT	max_depth = 20
	criterion = gini
KNN	n_neighbors = 9
	metric = minkowski
	algorithms = auto
	weights = distance

**Table 4:** Classifier results with considered metrics for two classes.

Model	Accuracy	Precision	Recall	F1
RF	0.858	0.854	0.773	0.846
LR	0.841	0.823	0.714	0.811
SVM	0.829	0.768	0.718	0.773
DT	0.810	0.813	0.764	0.809
KNN	0.821	0.824	0.745	0.814

**Table 5:** Classifier results with considered metrics for three classes.

Model	Accuracy	Precision	Recall	F1
RF	0.824	0.847	0.784	0.832
LR	0.810	0.814	0.706	0.799
SVM	0.817	0.759	0.732	0.768
DT	0.802	0.804	0.752	0.798
KNN	0.813	0.818	0.738	0.806

**Table 6:** RF results with considered metrics for two classes.

Classes	Accuracy	Precision	Recall	F1	Specificity
No Stress	0.818	0.814	0.751	0.834	0.773
Stress	0.899	0.894	0.796	0.859	0.832

**Table 7:** RF results with considered metrics for three classes.

Classes	Accuracy	Precision	Recall	F1	Specificity
No Stress	0.797	0.844	0.781	0.826	0.794
Stress 1	0.802	0.841	0.785	0.830	0.797
Stress 2	0.877	0.853	0.788	0.843	0.803

**Table 8:** Classifier results in terms of the specific tasks.

Model	Task 1	Task 2	Task 3	Task 4
RF 2 classes	0.882	0.921	0.891	0.913
RF 3 classes	0.801	0.822	0.813	0.821

**Table 9:** Classifier results with considered metrics for two classes using a leave-one-subject-out cross-validation.

Model	Accuracy	Precision	Recall	F1	Specificity
RF	0.847	0.846	0.775	0.838	0.852
LR	0.832	0.819	0.709	0.808	0.739
SVM	0.824	0.759	0.713	0.769	0.782
DT	0.806	0.811	0.758	0.802	0.774
KNN	0.819	0.817	0.739	0.808	0.738

**Table 10:** Classifier results with considered metrics for three classes using a leave-one-subject-out cross-validation.

Model	Accuracy	Precision	Recall	F1	Specificity
RF	0.817	0.836	0.779	0.817	0.788
LR	0.805	0.806	0.701	0.786	0.751
SVM	0.808	0.751	0.726	0.763	0.756
DT	0.794	0.793	0.749	0.793	0.792
KNN	0.801	0.804	0.726	0.798	0.769

**Table 11:** RF results with considered metrics for two classes using a leave-one-subject-out cross-validation.

Classes	Accuracy	Precision	Recall	F1	Specificity
No Stress	0.843	0.843	0.773	0.836	0.849
Stress	0.852	0.851	0.776	0.841	0.854

**Table 12:** RF results with considered metrics for three classes using a leave-one-subject-out cross-validation.

Classes	Accuracy	Precision	Recall	F1	Specificity
No Stress	0.811	0.829	0.778	0.813	0.781
Stress 1	0.815	0.838	0.777	0.814	0.784
Stress 2	0.823	0.841	0.784	0.822	0.802

## Data Availability

Dataset available on request from the authors.
